# Burst abdomen: a preventable risk of severe maternal morbidity in a developing country (a case-control study at a university teaching hospital in Tanzania)

**DOI:** 10.11604/pamj.2024.48.64.39044

**Published:** 2024-06-19

**Authors:** Andrew Hans Mgaya, Salim Alli Maumba, Bosco Pius Mapunda, Sophia Isaac Kiwango, Raymond Thomas Kiponza, Nathanael Luther Mtinangi

**Affiliations:** 1 Department of Obstetrics and Gynaecology, Muhimbili National Hospital, PO Box 65000, Dar es Salaam, Tanzania,; 2 Department of Women´s and Children´s Health/International Maternal and Reproductive Health and Migration, Uppsala University, Uppsala, Sweden

**Keywords:** Burst abdomen, wound dehiscence, surgical site infection, abdominal fascia closure, caesarean section

## Abstract

**Introduction:**

burst abdomen is a preventable complication of caesarean section that carries an increased risk of maternal death, especially in developing countries including Tanzania. The study aimed to identify the risk factors and high-risk patients for burst abdomen at Muhimbili National Hospital in Tanzania.

**Methods:**

a case-control study was performed at Muhimbili National Hospital in Dar es Salaam from 2^nd^ April to 27^th^ December 2019. Characteristics of interest of one case of burst abdomen were compared to three randomly selected controls that consisted of caesarean deliveries either 24 hours before or after the time of delivery of cases. The chi-square test, Fischer´s exact test, and multivariate analysis were used. The level of significance was p < 0.05.

**Results:**

a total of 524 women that met the inclusion criteria, comprising 131 cases and 393 controls, delivered by caesarean section in the most recent pregnancy at Muhimbili National Hospital. Cases were independently associated with perioperative illness, including cough (OR 3.8, 95%CI 1.9-7.6), chorioamnionitis (OR 4.5, 95% CI 1.3-14.7), and surgical site infection (OR 3.2, 95% CI 1.7-6.4), and a vertical midline incision wound (OR 1.9, 95% CI 1.2-3.1) compared to control group. Most cases (70%) had intact sutures and loose surgical knots.

**Conclusion:**

burst abdomen remains a cause of unnecessary severe maternal morbidity and is independently associated with perioperative illnesses such as cough, chorioamnionitis surgical site infection, and a vertical midline abdominal incision. Thus, there is a need for modifying abdominal fascia closure techniques for patients at risk.

## Introduction

Burst abdomen implies postoperative wound separation of the fascia that can subsequently manifest with skin and subcutaneous tissue disruption, hence also referred to as complete wound dehiscence [1]. Over decades, burst abdomen has been a frustrating but preventable complication [2,3] commonly following emergency laparotomy [4,5], through a midline incision [6,7], including the caesarean section (CS) [8,9]. Burst abdomen after CS demands immediate intervention to combat substantial risks of severe maternal morbidity and death [10-12]. The overall incidence of burst abdomen is as high as 30% [3,13] in developing countries, compared to the low incidence of 0.4% to 3.5% in developed countries for high-risk patients [14,15]. Maternal infection is a risk [9] and also a complication [9,11] of a burst abdomen, especially in developing countries including Tanzania [16,17]. Therefore, identifying predictors of wound dehiscence ought to enable appropriate selection of abdominal closure techniques, in order to prevent or at least reduce the incidence of burst abdomen in the context of developing countries.

Burst abdomen usually presents before the 14^th^ postoperative day [16] and more commonly on the 5^th^ to 8^th^ day after abdominal surgery. Wound healing progressively increases wound strength up to 20% from the first day to the third week, and the wound finally reaches 70%-80% strength in the 6^th^ to 12^th^ weeks [11]. Complete wound dehiscence is a consequence of impeded wound healing [13,18], precipitated by increased intra-abdominal pressure causing tension on the suture that can cause stitches to either tear off or cut through from the abdominal fascia, or else loosen the surgical suture knots. Thus, inappropriate surgical suturing techniques significantly contribute to wound failure and subsequent burst abdomen [19]. Evident patients´ related predictors of post-CS burst abdomen include perioperative infections from prolonged labour and chorioamnionitis [9,11], perioperative illnesses such as cough [10,11] and respiratory distress [10,11], and other conditions contributing to delayed wound healing with or without increasing intra-abdominal pressure. These conditions include, but are not limited to diabetes mellitus [20], hypertension [20], jaundice [6,20], uremia [20], ascites [13,21], anaemia [20], abdominal malignancy [6,18] and obesity [21] that were controverted by other studies [3,4,7,11]. Hence, it was important to determine the risk factors of wound dehiscence based on the local structure and practice of care and the health status of the specified patient population.

Our Centre, Muhimbili National Hospital (MNH) [22], is a tertiary referral facility that performed five to twenty-five CSs daily, where two obstetrics and gynaecology registrars or residents perform most of the operations during a 24-hour work shift. The operating theatre registry revealed a three times increase in burst abdomen repair surgeries in three years from 2013, and at least 25 cases of abdominal repair were recorded by the middle of 2017, of which 40-50% were from caesarean deliveries performed at MNH. As a teaching hospital, MNH ought to impart correct surgical skills to care providers and trainees from numerous institutions in and outside Tanzania, and advocate evidence-based clinical practice in health facilities within the health system. Therefore, we aimed to identify risk factors and high-risk patients for developing burst abdomen at MNH, a university teaching hospital in Tanzania.

## Methods

**Study design and period:** a case-control study was performed at Muhimbili National Hospital (MNH) from 2^nd^ April to 27^th^ December 2019. Cases of burst abdomen following caesarean delivery at MNH from January 2014 to December 2018 were identified. Characteristics of interest of each identified case were compared to those of three controls, which consisted of caesarean deliveries either 24 hours before or after caesarean deliveries of cases; and thereafter, discharged without signs of burst abdomen.

**Study settings:** Muhimbili National Hospital is a tertiary referral health facility serving the city of Dar es Salaam and neighbouring regions including Lindi, Mtwara, Pwani, Zanzibar and Morogoro. As a teaching university hospital, MNH also serves as a medical training facility for numerous universities in Tanzania. Like other public health facilities, the cost of maternity service at MNH has user-fee exemption and cost-sharing modalities [22,23] for clients who were referred from public hospitals. Self-referred clients and referrals from private health facilities are received as private clients under intramural private practice management (IPPM) either as health-insured or clients who pay services in cash. Maternity services for public and private clients are comparable, except that private clients have the privilege of choosing specialist services and receiving more comfortable in-patient accommodation in dedicated private wards.

Obstetric and newborn care at MNH takes place in two maternity buildings. Maternity one building is mostly for antenatal and postnatal in-patient service for cost-sharing and user fee-exempted clients. Other functions in the maternity one building include sonographic imaging services, pharmacy services, and in-patient registration and cashier counters for the two maternity buildings. Maternity two building consists of a reproductive and child health (RCH) clinic, in-patient wards for antenatal and postnatal mothers under IPPM and nursing mothers performing Kangaroo Mother Care. The operating theatre building is near maternity buildings. There are four operating rooms capable of accommodating major and minor surgeries under local, regional and general anaesthesia. Routinely two out of four operating rooms were designated for obstetric procedures that were mostly CSs. There are two working shifts for nurses and other clinical support staff. For doctors, one consultant, one specialist and two registrars or residents are on call for 24 hours.

The annual rate of delivery was 8,600 in 2010 to 9,000 in 2020 for both public (60%) and private (40%) clients at a rising CS rate of 40% - 54% for the past 10 years. Most of CSs were performed by obstetricians and obstetric registrars/residents; although, the decision for surgery was commonly made in consultation with obstetricians. Perioperative antibiotics including intravenous Ceftriaxone and Metronidazole were routinely administered to all patients for CS [24]. Generally, a sub-umbilical midline incision was more commonly performed for emergency CSs compared to elective CSs when Pfannenstiel-transverse incision was used. Spinal anaesthesia was preferable compared to general anaesthesia unless there was a contraindication of the former. Routinely, the rectus fascia closure was by continuous suturing technique using *“Polyglactin 910 suture”* or *“Polypropylene suture”* of size number 1. Postoperatively, CS deliveries were discharged on the second or third day, except when there were maternal complications or the newborn fell sick. Wound inspection was usually performed before the patients were discharged. On average most of the burst abdomen cases were diagnosed from 7^th^ day, postoperatively.

Tanzania is a low-middle income country with the largest business city of Dar es Salaam, which is inhabited by over 6 million people, where MNH is located. The health system pyramid puts MNH at the apex of an inclusive network of mainly government-owned health facilities organized in such a way that dispensaries and health centres serve most of the population as primary health care facilities, while district, regional and specialized/consultant hospitals serve as referral health facilities. All levels of public and some private health facilities provide RCH services, including antenatal and postnatal care, under-five care, family planning counselling and provision of contraception, essential vaccinations services, HIV/AIDS counselling and testing, and emergency obstetric and newborn care (EmONC)

### Study population and sampling technique

**Eligibility and data sources for case:** all cases of burst abdomen repair were identified from the operating theatre registry. Thereafter, the place where prior CS delivery was performed was verified using midwifery registry, before the participants were listed. Clinical notes of the listed cases were retrieved from medical records and obstetric and newborn databases, and thereafter the inclusion criteria were applied. The inclusion criteria were presence of doctors´ notes, pre-operative checklists and operation notes at the time of admission for burst abdomen repair and the time of previous CS performed at MNH.

**Eligibility and data sources for controls:** the date and time of the previous CS of the case were used to identify potential controls including all patients who delivered by CS within 24 hours either before or after CS delivery of the cases. The controls were identified from the operating theatre records and verified from the midwifery registry before being listed. Clinical notes of the listed controls were retrieved from medical records, and the inclusion criteria were applied. The inclusion criteria of control were similar to that of the cases, except that controls did not have any recorded clinical features of wound dehiscence at the time of discharge. Three eligible controls were randomly selected using computer-generated random numbers based on a total number of all potential eligible controls (5 to 21 parturients) that were delivered either 24 hours before or after the CS of cases. The process of recruitment continued until the required sample size was achieved.

**Sample size determination:** the sample size was calculated using Epi info 7 TM assuming that the percentage of exposure in the control group was 50% as it was unknown; the least extremely detected odds' ratio was 2; the power of the study (1-β) was 80%; the confidence level (CI) was 95% and the ratio between case to control was 3. The required sample size was 100 cases compared to 300 controls.

### Data collection

**Data collection tool and study variables:** data were collected from clinical notes and supplemented by clinical information from the operating theatre registry, midwifery registry and obstetric and newborn registry when applied. A standard questionnaire with three sections was the data collection tool that inquired background characteristics during admission for previous CS including age, date and time of admission, admission category (referral or non-referral/public or private category) and parity on the first section. The second section inquired about risk factors associated with previous CS, such as documented obstetric complications including obstructed labour/prolonged labour, chorioamnionitis, postpartum hemorrhage (i.e. for estimated blood loss > 1000mls), the specified amount of blood loss and documented conditions such as placenta previa/accrete, abruption placenta, eclampsia, chronic hypertension, HELLP syndrome, Heart failure, Intra-operative visceral injury, abdominal malignancy and presence of uterine scar, and number and types of previous abdominal surgical scars.

Other variables also included documented symptoms or outcomes of peri-operative illnesses including cough, respiratory distress, constipation, vomiting, anaemia, ascites, jaundice, admission to highly dependent ward/intensive care unit (ICU) and presence of surgical site infection (SSI). Other additional variables included documented immunosuppressive conditions or treatment such as Diabetes Mellitus, HIV infection, blood disorders (required to mention e.g. leukemia, lymphoma and thrombocytopenia) and use of steroids or cytotoxic therapy. The third section inquired about the date and time of postoperative diagnosis of burst abdomen, intra-operative findings and outcome of abdominal wall repair (for cases only). The variables of interest included type and integrity of abdominal rectus fascia suture, absence or presence of SSI and maternal outcome including blood transfusion, peri-partum hysterectomy, admission to ICU and maternal death. Questionnaires were examined for completion before data entry. There was no missing data.

**Definition of terms:** perioperative illnesses were defined based on what was documented in the clinical notes during admission for previous CS, which in this case was the most recent caesarean delivery. Anaemia was defined in three categories based on recorded Haemoglobin (Hb) levels: mild anaemia (Hb levels from 10g/dl to 11.9g/dl), moderate anaemia (Hb level from 7g/dl to 9.9g/dl) and severe anaemia (Hb levels less than 7 g/dl) [25,26]. Surgical site infection was defined either as documentation of wound infection/sepsis or characteristics of the surgical wound including the presence of pus discharge/redness/slough [27]. Timing of prior CS was categorised as during work hours (0800-1600hrs) and off hours (1601-0759). Clinically significant use of steroids, cytotoxic drugs and other immunosuppressants was considered when documented in the clinical notes.

**Data analysis:** data was entered and analyzed using SPSS ver. 23 (IBM SPSS, Chicago, IL). Data entry and cleaning included amending information that was incomplete or suspected to be incorrect by cross-checking the patient records from the obstetric and neonatal database [23] and ward round registers. Typographic errors and duplicated information were removed. We analysed the difference in the percentage of cases compared with controls based on background characteristics, obstetric risk factors peri-operative illnesses and patient-related factors using Pearson´s Chi-square test or Fischer´s exact test, when appropriate. The level of significance (α) was p < 0.05. When there was a significant difference in the percentage of characteristics of interest in cases compared to controls by Chi-square test and Fischer´s exact test, a multivariate regression analysis was performed to determine independent association using Odds ratio (OR) with 95% confidence intervals. Simple descriptive statistical analyses were used to present the percentage distribution of characteristics and outcomes of cases of burst abdomen. The study results were tabulated.

## Results

A total of 524 women who met the inclusion criteria were assessed (Table 1). The studied group composed of 131 cases and 393 controls the majority were 20-34 years old. The median age (range) was 29 (14-45) years and cases were older than the controls (30(16-45) vs. 29(14-47) years; p=0.03). The majority of the studied group had 2 to 4 deliveries (55%). Parity was comparable between the cases and control groups (all p=0.37). Nearly three-quarters of the studied group was composed of referral patients. There was a higher percentage of cases that were referrals when compared to the control group (81% vs. 71%; p=0.02). The majority of the studied group (70%) was composed of patients who were exempted or partially paid for service under the national health policy. Nonetheless, the percentage of cases and control within the payment category, level of emergence (elective vs. emergency CS), timing of CS (work hours vs. off-hour), and provision of perioperative antibiotics were comparable (all p>0.06).

**Table 1:** percentage difference in background characteristics of the studied groups

Characteristics	Total		Cases		Control		p-value
	N=524	%	n=131	%	n=393	%	
Age (years)							0.03
< 20	32	6.1	6	4.6	26	6.6	
20 - 34	375	71.6	89	67.8	286	72.8	
35 -39	82	16.8	22	17.3	60	15.6	
≥40	35	6.5	14	10.7	21	5.3	
Parity							0.37
1	192	33.6	44	33.6	148	37.7	
2-4	286	54.6	78	59.5	208	52.5	
≥ 5	46	8.8	9	6.9	37.0	9.4	
Admission status							0.02
Referral	384	73.3	106	80.9	278	70.7	
Non-referral	140	26.7	25	19.1	115	29.3	
Payment category							0.06
Exempted or cost-sharing	381	72.7	107	81.7	247	69.7	
Full cost - cash or credit payment	92	17.6	15	11.5	77	19.6	
NHIF coverage	51	6.9	9	6.9	42	10.7	
Level of urgency							
Emergency	443	84.5	110	84	333	84.4	0.83
Elective	81	15.5	21	16	60	15.3	
Timing of surgery							
Work hours	202	38.5	42	32.1	160	40.7	0.08
Off hours	322	61.5	89	67.9	233	59.3	
Provision of perioperative antibiotics							
Yes	503	96.0	125	95.5	378	96.2	0.7
No record	21	4.0	6	14.4	15	3.8	

A higher proportion of cases had obstructed/prolonged labour (61% vs. 13%) and choriamnionitis (6.1% vs. 1.3%), compared to the control group (all p<0.01) (Table 2). Inversely, there were lower proportions of cases with previous uterine scars, compared to their counterparts (17% vs. 29%;p=0.002). The proportion of cases and control for other obstetric risk factors, including excessive blood loss, hypertensive disorders, HELLP syndrome, or Heart failure, was comparable. A higher proportion of cases had perioperative respiratory distress (4% vs. 0.2%) and cough (21% vs. 7%), compared to the control group (all p<0.001). The majority of the studied group (87%) had anaemia. Specifically, a higher proportion of cases had moderate (35% vs. 20%) and severe anaemia (13% vs. 5%), and wound sepsis (14% vs. 6%), compared to the control group (all p≤0.001). The majority of the studied groups had a vertical (60%) rather than a transverse surgical abdominal scar (28%). Nonetheless, a higher proportion of case had a vertical abdominal incision from previous CS, compared to the control group (74% vs.55%;p=0.001).

**Table 2:** percentage difference in obstetric risk factors and perioperative illness of the studied groups

Risk factors	Total		Cases		Controls		P-value
	N=524	%	n=131	%	n=393	%	
Obstructed /prolonged labour	118	22.5	80	61.4	38	13.0	<0.001
Chorioamnionitis	93	2.5	8	6.1	5	1.3	0.008
Postpartum hemorrhage	40	7.6	12	9.2	28	7.1	0.45
Amount of blood loss							
<500mls	45	8.7	8	6.3	37	9.5	0.07
500mls to 999mls	431	84.3	111	87.5	326	84.3	
1000mls to 1499mls	21	4.1	7	5.5	14	3.6	
1500mls to 1999mls	17	13.3	3	2.4	14	3.6	
≥ 2000mls	2	0.4	2	0.4	0	0	
Hypertensive disorders	19	3.6	6	4.6	13	3.3	0.5
HELLP syndrome	7	1.5	2	1.5	5	1.3	0.82
Heath failure	4	0.8	1	0.8	3	0.8	1
Previous uterine scar	134	25.6	22	16.8	112	28.5	0.002
Respiratory distress	8	1.8	6	4.0	2	0.2	<0.001
Cough	55	10.5	27	20.6	28	7.1	<0.001
Constipation	7	1.3	1	0.8	7	1.5	0.51
Vomiting	13	2.5	6	4.6	7	1.8	0.448
Perioperative anaemia							
No anaemia	71	13.6	15	11.5	56	14.6	<0.001
Mild anaemia	288	55.2	53	40.8	235	59.9	
Moderate anaemia	125	23.9	45	34.6	80	20.4	
Severe anaemia	38	7.3	21	13.1	17	5.4	
Ascites	9	1.7	4	3.1	5	1.3	0.448
Jaundice	16	13.1	7	5.3	9	2.3	0.079
Wound sepsis	46	8.8	23	13.6	23	5.9	0.001
Diabetes mellitus	24	4.6	8	6.1	16	4.1	0.33
HIV infection	36	6.9	11	8.4	25	6.4	0.42
Use of steroids	8	1.5	2	1.5	6	1.5	1
Intra-operative visceral injury	1	0.2	1	0.8	0	0	0.83
Abdominal tumor	1	0.3	1	0.3	0	0	0.25
Previous abdominal surgical scars							
None	388	74	108	82.4	280	71.2	0.09
One	118	22.5	20	15.3	98	24.9	
Two	17	3.2	3	2.3	14	3.6	
Equal or more than 3	1	0.2	0	0	1	0.3	
Types of abdominal surgical scar							
Vertical midline incision	314	59.9	98	74.8	243	61.8	0.007
Transverse incision	190	40.1	33	25.2	150	38.2	

The proportion of cases with perioperative constipation, vomiting, ascites, jaundice, Diabetes Mellitus, HIV infection, intraoperative visceral injury, and abdominal tumor was comparable to their counter parts (all p>0.09). Additionally, a similar proportion of cases and controls used steroids and had the same number of previous abdominal surgical scars (all p>0.07). None of the women smoked or used cytotoxic or other immunosuppressive therapies in the study group. Multivariate analyses of patient-related risk factors (Table 3) showed an independent association of burst abdomen cases with chorioamnionitis (Odds ratio, OR 4.5, 95% CI 1.3-14.7), cough (OR 3.8, 95%CI 1.9-7.6), wound sepsis (OR 3.2, 95% CI 1.7-6.4) and a vertical midline incision scar (OR 1.9, 95% CI 1.2-3.1), compared to controls. Burst abdomen cases were less likely to have a previous uterine scar, compared to the control group (OR 0.4, 95%CI 0.2-0.6).

**Table 3:** bivariate and multivariate logistic regression analysis of risk/likelihood of burst abdomen by patient-related perioperative illness and type of previous abdominal scar of the studied groups; odds ratio (OR) and 95% confidence interval (CI)

Risk factors	Cases n=131(%)	Controls n=393(%)	Unadjusted OR(95%CI)	Adjusted OR(95%CI)
Obstructed/prolonged labour				
Yes	80(61.1)	38(9.6)	14.2(8.7-24.5)	1.2(0.7-1.9)
No	51(38.9)	355(72.8)	1	
Chorioamnionitis				
Yes	8(6.1)	5(1.3)	5.1(1.4-19.9)	4.5(1.3-14.7)
No	123(83.9)	388(98.7)	1	
Previous uterine scar				
Yes	22(16.8)	112(28.5)	0.51(0.29-0.86)	0.4(0.2-0.6)
No	109(83.2)	281(71.5)	1	
Respiratory distress				
Yes	6(4)	2(0.2)	9.4(1.6-95.7)	4.1(0.7-23.8)
No	125(96)	391(98)	1	
Cough				
Yes	27(20.6)	28(7.1)	3.4(1.8-6.2)	3.8(1.9-7.6)
No	104(79.4)	365(92.9)	1	
History of anaemia				
No anaemia	15(11.5)	56(5.9)	1	
Mild	53(40.8)	235(59.9)	0.8(0.4-1.7)	
Moderate	45(34.6)	80(20.4)	2.1(1.1-4.2)	1.0(0.5-4.4)
Severe	21(13.4)	17(5.4)	4.6(1.8-11.9)	1.7(0.7-4.4)
Wound sepsis				
Yes	23(13.6)	23(5.9)	3.4(1.8-6.7)	3.2(1.7-6.4)
No	108(86.4)	370(94.1)	1	
Types of abdominal scar				
Vertical midline incision	98(74.8)	243(61.8)	2.9(1.2-2.9)	1.9(1.2-3.1)
Transverse incision	33(25.2)	1150(38.2)	1	

Clinical findings of burst abdomen cases revealed the use of polyglactin 910 sutures (37%) and polypropylene sutures (34%) for closure of abdominal fascia. Most of the cases presented completely gapped wounds with intact sutures (70%) that were loosely binding wound margins (62%) with or without a loosened surgical knot. Half of the cases were not reported to have SSI and three-quarters of the cases presented on the 7^th^ day or later after the previous CS. Maternal outcomes of burst abdomen cases included blood transfusion (50%) peripartum hysterectomy (23%) and admission to ICU (12%). Nine out of 131 cases died. The documented reason for maternal death was septicemia (3), severe anemia (1), pre-eclampsia/HELLP syndrome (3), and peripartum cardiomyopathy (2). The length of hospital stay for surviving cases was an average of 8 days of which the shortest stay was 2 days while the longest stay was 78 days.

## Discussion

We found burst abdomen was predictable and a cause of severe maternal morbidity that was also associated with maternal death following CS. Patient-related risk factors such as a vertical midline abdominal incision, perioperative infections - chorioamnionitis and SSI, and cough during CS were independently associated with burst abdomen in our study population. In our study, severe maternal complications and near misses such as anaemia, pre-eclampsia/HELLP syndrome, and peripartum cardiomyopathy increased the risk of maternal death among burst abdomen cases. Further, post-CS burst abdomen was associated with prolonged hospital stay, blood transfusion, and admission to the intensive care unit (ICU) that imposed unnecessary cost burdens on the family and health system [28]. Similar to other studies [19,29, 30], SSI was primarily a cause or secondarily an effect of a burst abdomen. Together or independently, SSI and chorioamnionitis were independent risks of burst abdomen, despite the use of pre-operative antibiotics in more than 95% of the studied group. Maternal infection associated with a burst abdomen included deep SSI such as necrosis of the uterine incision site that contributed to peri-partum hysterectomies, blood transfusion, and admission to ICU. During previous CS, most of the cases (80%) were referred to MNH from another public hospital; therefore, efforts to prevent burst abdomen require multilevel measures of infection prevention control (IPC) from the health facility at the first contact with patients and within the referral system.

The increased risk of a burst abdomen due to peri-operative cough was probably due to impulsively increased intra-abdominal pressure on a vertical midline abdominal surgical wound on the first to second week after CS, when the wound was at most with 20% strength [11]. Peri-operative vomiting and constipation did not have the same effect, perhaps because of the small number of participants presenting with the peri-operative characteristics of interest in the studied group. The level of anaemia was also comparable between cases and controls, even though anaemia presented a risk of poor wound oxygenation that could have led to delayed wound healing. The overall high prevalence of anemia in the study group (80%) could have obscured detecting the difference in odds of anaemia between cases and controls. On the other hand, the high prevalence of anaemia should not go unnoticed, as it reflects the poor nutritional status of pregnant women and possible inadequacies of prenatal care.

The proportion of cases with multiple abdominal scars, hypertension, diabetes, and malignancy was comparable with that of controls, contrary to other studies [11,13,18]. However, in Tanzania, pregnancy-related hypertensive disorders are one of the main causes of hospital maternal death [31,32] and diabetes increases the risk of obstetric hemorrhage and hypertension [33] indirectly contributing to severe maternal morbidity [34]. Therefore, skillfully performed CS wound close is demanded to avoid the risk of wound failure [35] that may subsequently increase the odds of maternal death among hypertensive and/or diabetic parturients.

Considering that the majority of cases had complete wound dehiscence with unbroken sutures that were either avulsed from the rectus fascia margin or loosely bound across the skin, subcutaneous tissue, and fascia edges; it was likely that the abdominal closure technique was substandard. Consequently, the observed trend of occurrence of burst abdomen from 2013 to 2018 was inevitable. On the other hand, despite advancements in pre-operative care, surgical techniques, and manufacturing of suture materials, the incidence of burst abdomen has not significantly changed over the past decades [36,37], especially in developing countries. The reason for the subtle change might be due to the increasing incidence of risk factors within the patient population and/or unchanged surgical technique and clinical practice. Randomized controlled trials and meta-analyses have recommended optimal fascia closure in emergency and elective laparotomies [38,39], in addition to basic surgical training dictum of 1cm “bites” followed by 1 cm of progress [40]; and a decades-long concept of an optimal suture length to wound length ratio of approximately 4: 1 [41,42], which probably was not routinely applied during CSs of our case. Previous evidence of comparable outcomes between interrupted and continuous wound closure [37,42] underpinned the importance of meticulous identification of fascial margins and suturing adequate bites from the fascia margins. Patients with an increased risk of wound dehiscence, in this case maternal infection and perioperative cough could have benefited from a modified suturing technique by employing interrupted X-suture [19], Smead-Jones suture [43] or application of plastic tube tensions suture [8]. As observed in previous studies [44,45], our findings continue to challenge teaching and clinical staff to recognize the gap and take prompt action in teaching, supervision, and mentoring of medical residents and junior staff, in order to maximize the opportunity of surgical skills transfer from specialists to junior medical doctors and residents.

**Study strength and limitations:** in this study, we compared the risk of a burst abdomen under case to control ratio of 1: 3 for randomly selected controls, to increase the power of the study and minimize the risk of selection bias. The time at which CS was performed for cases and controls was closely matched within a 24-hour margin, to minimize the confounding effect of the difference in the quality of care due to unaccounted changes of the structure process of care, and work environment [46]. Nonetheless, the effect of a possible change in quality of care during the day-work shift and night-work shift was accounted for during the analysis. The study findings can be generalized in health facilities that provide CEmOC and university teaching hospitals in developing countries, where there is still room for improving maternal and newborn care and surgical skills training. However, our findings had limitations including a risk of observer bias from using clinical notes as the main data source that varied in quality of documentation, and at times different data sources were used to supplement missing information. Further, even though the definition of terms were evidence-based it is difficult to ascertain the effect of the degree of illnesses such as cough, vomiting, and SSI to the outcome of interest. Retrospective data collection might have introduced selection bias of cases and controls because of missing or wrongly recorded information such as the date and/or time of previous CS. Our study did not assess surgical competence and the influence of work environment on adherence to standards of practice during CS, which were beyond the scope of this study.

## Conclusion

Burst abdomen remains a cause of unnecessary severe maternal morbidity and is independently associated with perioperative illnesses such as cough, chorioamnionitis surgical site infection, and a vertical midline abdominal incision. Thus, there is a need for modifying abdominal fascia closure techniques for patients at risk. The study findings provided room for further improving evidence-based clinical and surgical practice in peri-operative care in developing countries. Based on the study findings, we recommend that: post caesarean section burst abdomen should remain a quality indicator for maternal health care. Quality improvement measures at MNH and the Dar es Salaam health system should continue to address post-cesarean complications, and continuously upgrade the standards of CEmONC and IPC. Further studies are needed to objectively determine the effectiveness of surgical skills training for postgraduate residents, and assess opportunities and challenges of surgical skills transfer to junior medical doctors in university teaching hospitals such as MNH.

### What is known about this topic

- *Burst abdomen after CS presents a higher risk of severe maternal morbidity and death in developing compared to developed countries;*
- *Poor surgical techniques and perioperative illness including infections from prolonged labour and chorio-amnionitis, cough and respiratory distress, and other conditions; contributing to delayed wound healing significantly contribute to wound failure and subsequent burst abdomen*.

### What this study adds

- *Burst abdomen remains a preventable cause of unnecessary severe maternal morbidity following caesarean section; prevention of post-CS burst abdomen ought to reduce maternal morbidity and death; thus, post-CS burst abdomen events should be recognized as a quality of care indicator, so that efforts for purposefully identifying patient populations at risk and prevailing risk factors are made at a local level;*
- *Poor surgical techniques with or without pre-operatively evident risk factors of wound dehiscence significantly contribute to wound failure and subsequent burst abdomen; therefore, there is an opportunity to improve supervision and mentoring when performing CS, which is the most commonly performed major abdominal surgery in obstetrics and gynaecology*.
